# Open Meter Duo: Low-Cost Instrument for Fluorimetric Determination of Cholinesterase Activity

**DOI:** 10.3390/s24061774

**Published:** 2024-03-09

**Authors:** Ondřej Keresteš, Juan Daniel Mozo, Miroslav Pohanka

**Affiliations:** 1 Military Faculty of Medicine, University of Defence, CZ-50001 Hradec Kralove, Czech Republic; 2 Faculty of Experimental Sciences, University of Huelva, 21071 Huelva, Spain; jdaniel.mozo@diq.uhu.es

**Keywords:** fluorimetry, remote sensing, portable, low cost, cholinesterase, Arduino, 3D printing

## Abstract

Environmental screening is essential due to the increased occurrence of harmful substances in the environment. Open Meter Duo (OMD) is an open-source field photo/fluorimeter that uses an RGB diode that imitates a color according to the selected wavelength and uses a UV LED from the security kit diode as an excitation light source. The prepared PCB shield with a 3D-printed aperture was connected to Arduino UNO R4 WiFi. This system was used for the fluorescent detection of cholinesterase activity with the indoxyl acetate method. Carbofuran—a toxic pesticide—and donepezil—a drug used to treat Alzheimer’s disease—were tested as model inhibitors of cholinesterase activity. The limit of detection of indoxyl acetate was 11.6 μmol/L, and the IC50 values of the inhibitors were evaluated. This system is optimized for wireless use in field analysis with added cloud support and power source. The time of analysis was 5 min for the fluorimetric assay and 20 min for the optional photometric assay. The time of field operation was approximately 4 h of continuous measurement. This system is ready to be used as a cheap and easy control platform for portable use in drug control and point-of-care testing.

## 1. Introduction

Pesticide use is already largely pervasive across the sector of agriculture. Using pesticides helps to produce enough food for the increasing population. However, when released into the environment, they can contaminate water, soil, and air, therefore posing risks to non-target organisms, such as aquatic life, birds, and mammals. Prolonged exposure to these chemicals can lead to a range of adverse effects, including bioaccumulation, decreased reproductive success, and even mortality. Continuous monitoring of pesticide levels in the environment can serve as an early warning system for potential contamination events or areas with elevated concentrations of these chemicals [1,2,3].

Acetylcholinesterase (AChE, E.C. 3.1.1.7) is an essential enzyme for the cleavage of acetylcholine in the nervous system and one of the most used enzymes in biosensors for pesticides (e.g., organophosphates and carbamates) and pharmaceuticals (e.g., a drug for Alzheimer’s disease treatment, donepezil) inhibiting cholinesterase [4,5].

The screening of the kinetic parameters of novel drugs is also a common use of AChE as a biorecognition element [5]. For the construction of biosensors, it is beneficial to use AChE from *Electrophorus electricus* in preliminary studies, due to its high similarity to human AChE [6].

Ellman’s assay [7] is the gold standard for the determination of cholinesterase activity in botany, ecotoxicology, military toxicology, and drug control [8,9,10]. However, alternative substrates for cholinesterase determination exist.

The indoxyl acetate assay is the second most established method for cholinesterase determination by the photometric approach. It has already been validated and efficiently used as an alternative to Ellman’s method to determine cholinesterase [8]. Cholinesterase has been thoroughly tested using this substrate and its derivates [8,11,12]. The principle of the reaction is simple: indoxyl acetate, resp. indoxyl hydrolyzed from indoxyl esters undergoes oxidation to indigo, a blue pigment. While the colorimetric determination of cholinesterase by the indoxyl acetate method is relatively more time-consuming for field assays [13], indoxyl acetate is still used for photogrammetry and colorimetry assays [12,14,15]. The important reason for this is its aforementioned stability [8].

Although it has historically been one of the substrates tested for its fluorescence [16], there are few works that propose artificial derivates of indoxyl to detect hydrolyzed substrates fluorimetrically [8,16,17].

Despite the fluorescence of indoxyl, low-cost instrumentation for the fluorimetrical determination of cholinesterase activity has not been tested. Instead of that, we can find outstanding work describing the fluorimetric analysis of organophosphate pesticides with Esterase-2 from *Alicyclobacillus acidocaldarius* and its application [18,19,20].

The relatively high prices of benchtop photometers and fluorimeters limit their accessibility for a potential user; open-source microcontroller-based instrumentation offers a cost-effective and user-friendly alternative. It could be more beneficial to construct devices for specific uses, instead of developing universal instruments. However, these universal systems are based on low-cost electronics (such as Arduino NANO or UNO boards) but use costly detectors to establish sensitivity to various analytes with different spectral characteristics [21,22,23,24]. Low-cost electronics designed for specific uses may help build a foundation for novel applications using the Internet of (Analytical) Things [25].

Considering the current state of knowledge, with reference to the text above, it is evident that an affordable wireless portable device tested for the fluorimetric determination of cholinesterase is still missing. We developed Open Meter Duo (OMD), an instrument that is simple to manufacture and capable of an indoxyl acetate assay in field conditions. It is based on Arduino UNO R4 WiFi and uses a low-cost detector. The excitation source was disassembled from a security ultraviolet flashlight. All electronics were mounted on a lab-made PCB shield. With an internet connection and its own power supply, it is a self-contained system which responds to instructions through communication with a remote server, and it works for at least 4 h. The overall price of the presented system is approx. USD 95.

The indoxyl acetate assay was tested using a fluorimetric approach, while carbofuran [26] and donepezil [27] were tested as model inhibitors. Carbofuran is a highly toxic broad-spectrum N-methyl carbamate pesticide. Despite strict regulations, it can still be found in wildlife [28,29].

## 2. Materials and Methods

### 2.1. Design and Assembly of the Open Meter Duo (OMD)

The 3D design of the Open Meter Duo box, lid, and apertures was made in Fusion 360 v2.0.18220 (Autodesk, San Rafael, CA, USA). Three-dimensional models were processed for printing with PrusaSlicer v2.6 (Prusa Research, Prague, Czech Republic). The printing resolution was 0.2 mm high layers and 15 % infill in the lightning pattern. Three perimeters were used for printing. Models were therefore printed with Prusa MK3S+, a fused filament fabrication (FFF) type of printer, with multimaterial upgrade v2 (MMU2, Prusa Research, Prague, Czech Republic). Acrylonitrile styrene acrylate filament (ASA, Prusa Research, Prague, Czech Republic) was used for the box and lid. Recycled polylactic acid filament (PLA, Smart Materials 3D, Alcalá la Real, Spain) was used to print the apertures. Prints from ASA need to be made in the box, and an at least 3 mm brim and skirt with the same height as the model need to be added to prevent damage to the model while printing. It is also possible to print the box in one color, because a 3D-printed box is prepared to insert the electronics in the same way as a drawer on a desk. The cuvette chamber could be replaced by a tube chamber. Both chambers are connected directly to a novel Arduino shield using standard M2 bolts and nuts.

Hardware for the system was assembled using electronics based on Arduino UNO R4 WiFi (Arduino, New York City, NY, USA), which is an often-used microcontroller board footprint [30]; a TSL230R light-frequency sensor (TAOS, Texas Advanced Optoelectronic Solutions, Plano, TX, USA) [31]; and a diffused RGB LED obtained from a local store. A flashlight included in the invisible marker set (Centropen, CZE) was used as a light source for the fluorescence assays [32]. The bill for the materials is included in Table 1.

**Figure 1:**
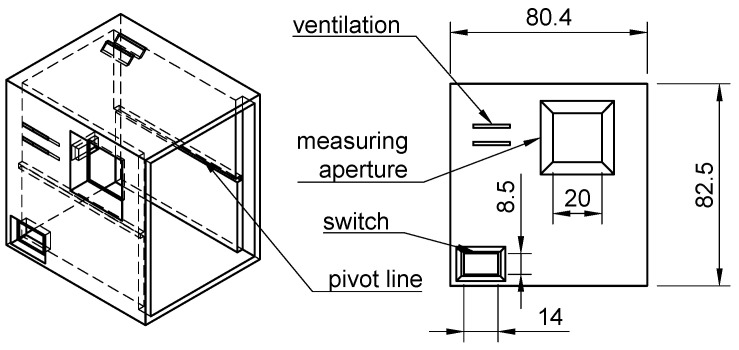
Overview of projections of OMD box.

**Figure 2:**
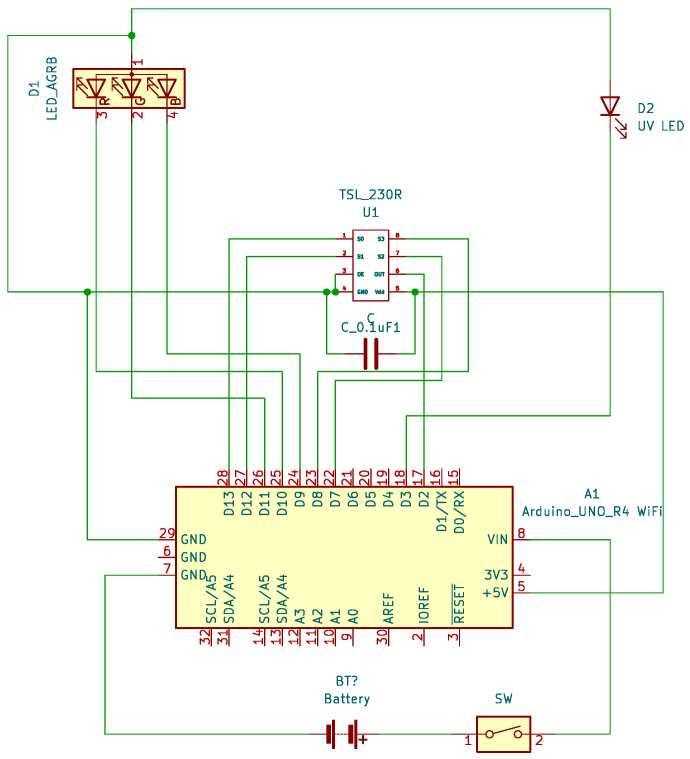
Schematic of the remote version of the Open Meter Duo system.

**Figure 3:**
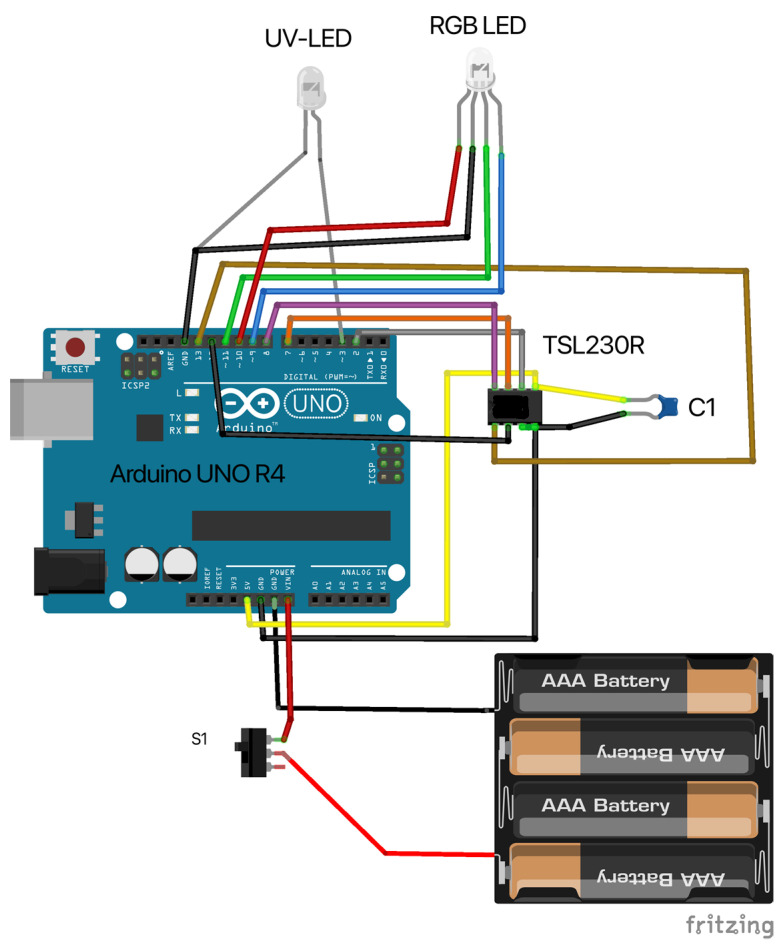
Wiring scheme of Open Meter Duo. Pin 1 on TSL230R sensor is connected to the brown wire.

The electronics were designed in open-source KiCad v6.0.6 software. The shape of the shield was chosen to enable us to insert the assembled electronics in the box along with pivot lines in the same way as a drawer on a desk (see Figure 1). Headers for the RGB LED and TSL230R were placed near one corner of the PCB to ensure that access to the detector and source was as close to the edge of the board as possible. The reason for this was to avoid potential fluid leakage into the electronics as much as possible. Therefore, when in use, the orientation of the shield plane was vertical. The schematics of OMD are depicted in Figure 2. The wiring scheme is depicted in Figure 3.

### 2.2. Characterization of Light Source

The RGB LED module and UV LED were connected to the Spectrovis Plus fiber optic module (Vernier, Beaverton, OR, USA) through a 3D-printed insert in the same way as shown in a recent study [33]. The light source used was programed with the implemented library for the translation of the desired wavelength to imitate a color according to the combination of information about color depth in the red, green, and blue channels.

The emission spectra of each LED (red, green, and blue) were measured separately by the Spectrovis Plus portable photo-fluorimeter (Vernier, Beaverton, OR, USA) with Logger Pro v3.16.2 software (Vernier, Beaverton, OR, USA).

### 2.3. Testing of the TSL230R Sensor

To obtain the lowest sensor response, a cuvette filled with distilled water was inserted into the OMD and covered with a 3D-printed plastic cap. The RGB LED was turned OFF. This condition was therefore acquired as a response to dark. The mean of the response and standard deviation were calculated for ten repetitions. The same was tested for the fluorimetry setup, but the UV light was turned ON to acquire the background intensity.

The testing of different light conditions was carried out by evaluating at least 30 response values for colors imitating wavelengths of 412 nm, 435 nm, 555 nm, 690 nm, and 700 nm. Sample values were set to 200 for the fluorimetric mode and 2000 for the photometric mode. The sensitivity was set to high.

### 2.4. Control of the Open Meter Duo

Arduino IDE software v2.2.1 (Arduino, New York, NY, USA) software was first used to upload the program and control OMD while optimizing its response. The intensity value of the light source, the wavelength value used to calculate the combination of color channels to emit the appropriate light, the frequency value, and the number of samples could be obtained by comma-separated values.

There are two options for controlling OMD. The first, offline, is based on the use of the Microsoft Excel Data Streamer add-in. The second is remote control through the Arduino cloud interface.

#### 2.4.1. MS Excel Data Streamer

The Data Streamer add-in in MS Excel was used to acquire the data for the final evaluation. The values of imitated wavelength, intensity of the source, output frequency, and sampling were acquired in one line. The time code was appended automatically to each line in Data Streamer.

#### 2.4.2. Arduino IOT Cloud

**Figure 4:**
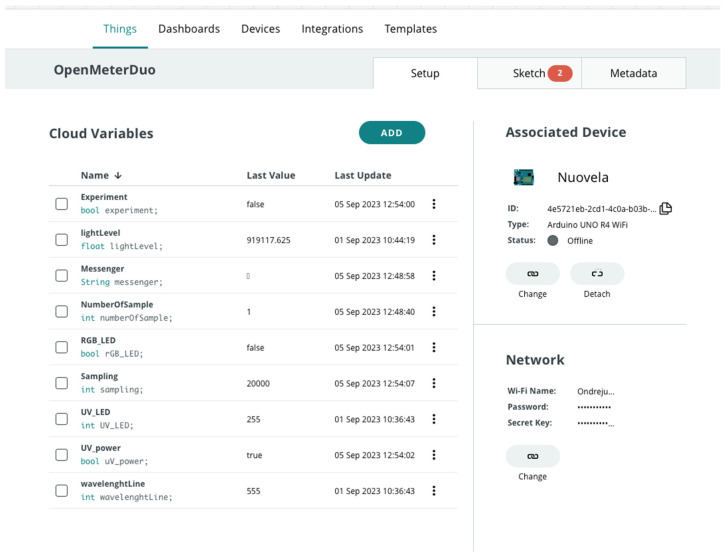
List of attributes needed for Open Meter Duo programming.

The Arduino cloud interface was tested for controlling OMD (more detailed steps are noted in [34]). It was essential to directly import the ‘WavelenghtToRGB’ library through a web platform and append it to the prepared code. See Figure 4 for an idea of the variables which could be used for work with OMD. Data acquisition could be controlled and customized by the dashboard on Arduino cloud. It starts automatically after the connection with the cloud is established. Users can edit further parameters in the dashboard. There are layouts for computer and mobile, which can be customized. Options for further dashboard customization are noted on the manufacturer’s website [35].

### 2.5. Acetylcholinesterase Solution

A standard enzyme sample of acetylcholinesterase from *Electrophorus electricus* (AChE, EC 3.1.1.7, type V-S, Merck, Darmstadt, Germany) was prepared by the solubilization of crude enzyme protein in phosphate-buffered saline (pH 7.4, PBS; Merck, Darmstadt, Germany) with the addition of bovine serum albumin (Merck, Darmstadt, Germany) as a stabilizer at a total concentration of 0.5 mg/mL (*w*/*v*). The activity of this concentrate was optimized to provide an enzymatic reaction with an optical density (OD) of 0.5 to 1 after incubation for 2 to 5 min. This mixture was transferred to microcentrifuge tubes and stored in a freezer (−20 °C).

### 2.6. Indoxyl Acetate Assay

#### 2.6.1. Spectral Characteristics of Indoxyl Acetate and Its Hydrolysis

In a standard fluorimetric cuvette, 100 μL samples of indoxyl acetate solutions diluted on a two-fold dilution scale (3.125, 6.25, 12.5, 25, 50, and 100 mmol/L in absolute ethanol) were added to a mixture of 75 μL of AChE concentrate and 2825 μL of PBS. Fluorimetric determinations of emission maxima were acquired in pentaplicate with the use of Spectrovis Plus after 1 h.

#### 2.6.2. Simple Fluorimetric Assay

To optimize the concentration of indoxyl acetate for the reaction, it was diluted in a two-fold series to a concentration of 3.125–100 mmol/L. The volume of each calibrant was 30 μL/3 mL of the reaction mixture in phosphate-buffered saline (PBS, pH 7.4). The final concentrations of indoxyl acetate in the cuvettes were 0.03125–1 mmol/L. The reaction was started by adding 75 μL of AChE concentrate, and the reaction was observed for 6 min. The initial value of the output frequency was subtracted as a blank value.

#### 2.6.3. Study of Fluorescence Stability

Next, 100 mmol/L and 50 mmol/L of indoxyl acetate calibrants were used to detect changes in the fluorescence signal over 1 h. The reaction mixture was identical to the one described above. The test was conducted by photogrammetry under UV light (λ_ex_ = 366 nm) with the use of our designed cuvette stand for 7 samples [36]. The stand was printed so that the same setup and material as a cuvette aperture could be used. Calibrants were tested in triplicate, and the seventh sample was a negative control without enzymes.

#### 2.6.4. Dual-Signal Concept

For the pilot study of the dual-signal option, measurements started in fluorescence mode. After the first five minutes of the ongoing reaction, photometric measurement was started. The reaction was continuously observed with live data streaming in the Data Streamer add-in. The aim of this part of the study was to show the opportunity of combining this assay with artificial intelligence modules written in high-level programming language in future. In this study, the fluorescence and photometry modes were evaluated separately.

The dual-signal approach was based on the change in mode between photometric and fluorimetric. The overall analysis time was 25 min. After 5 min of the fluorimetric assay, the photometric mode was activated for 20 min. The imitated color used for photometry was 620 nm; this was the peak emitted wavelength of the red LED in the RGB LED module. Unless the supposed wavelength for the indoxyl acetate assay was 670 [8], 605 [37], or 650 nm [38], 630 nm was suggested [12]. Due to the long time it took for the colored pigment to evolve, the photometric approach was only used for the demonstrative construction of the saturation curve.

#### 2.6.5. Determination of Model Cholinesterase Inhibitors

Donepezil hydrochloride was dissolved in phosphate-buffered saline solution (pH = 7.4) to a series of solutions with descendent concentrations. The highest concentration was 50 μmol/L, and the lowest was 0.078 μmol/L. The dilution of donepezil in 3 mL of reaction mixture was conducted by adding 75 μL of donepezil solution combined with 150 μL of AChE concentrate, which was filled up to 2970 μL with PBS in a cuvette. The final concentrations of donepezil in the cuvettes were 1.23–1250 nmol/L.

Carbofuran was dissolved in isopropyl alcohol to a 1 mmol/L stock solution. This solution was diluted to 25 μmol/L and therefore diluted again in a two-fold series. The smallest concentration of calibrant in the row was 0.049 μmol/L. The final concentrations of carbofuran were 1.95–625 nmol/L. Before the start of the reaction, 7 min of incubation time for carbofuran with 150 μL of AChE was allowed to establish the optimal inhibition effect, as reported in recent work [33].

The reaction was started by adding 30 μL of indoxyl acetate solution in absolute ethanol (50 mmol/L). It is not recommended to add indoxyl acetate solution in ethanol to a cuvette made from PMMA. It would damage the cuvette surface and interfere with the photo/fluorimetry assays. The degradation process is rather slow and therefore not significant in the first moments of analysis. This is the main limitation for future use.

The assay was evaluated by comparing the linear trends of the response to excitation with a UV source at a 90° angle to the detector.

### 2.7. Acquisition and Evaluation of Data

One approach is to set up OMD in the photometry regime. The second approach is to use the fluorometric regime, because the product of the hydrolysis of indoxyl esters emits light when excitated by UV light conditions [17,39,40].

#### 2.7.1. Spectrovis Plus

The measured value of fluorescence intensity was obtained at the start of the reaction, and this value was subtracted from the intensity value after the observation time. These values for each calibrant were therefore used for the construction of a saturation curve (different concentrations of substrate vs. fluorescence intensity after the observed time) The Michaelis–Menten nonlinear model was used for the evaluation of the enzymatic reaction. The calibration curve of carbofuran and donepezil was obtained by comparing the values of reached intensity with each inhibitor added in various concentrations to the reaction mixture.

#### 2.7.2. Open Meter Duo

For the indoxyl acetate fluorescence assay using OMD, reaction mixtures were measured for the time it took to acquire 1000 values by the Data Streamer add-in. In each case, it was at least 3 min. The final result values for slope comparison were obtained by the isolation of linear trends over a period of at least 2 min. This was the longest period until the increase in the signal became persistent. In particular, the high concentrations of the inhibitor caused a delay in onset. It was necessary to shift the period to avoid artificially lowering the slope and thus overestimating the difference between the inhibited and non-inhibited samples.

While evaluating the difference in output frequency, the values of the difference between the minimum and maximum values were obtained from GraphpadPrism v9.5.1 and compared to the total amount of acquisition time. The final results were calculated as a change per amount of time.

## 3. Results and Discussion

### 3.1. Design and Assembly of the Open Meter Duo (OMD)

**Figure 5:**
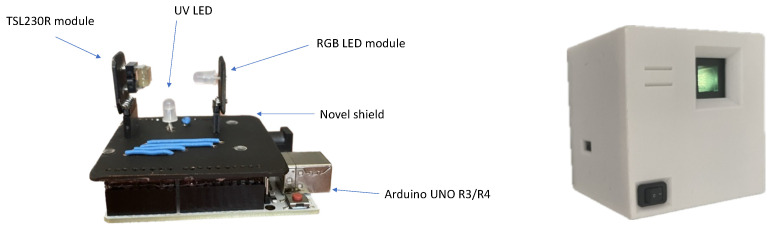
Overview of hardware (**left**) and system ready to use after installation (**right**).

Open Meter Duo (OMD)—a photo/fluorimeter based on a low-cost microcontroller, Arduino UNO R4 WiFi, a TSL230R sensor, and an RGB and UV LED—was prepared in this work. In Figure 5, an overview of the hardware and plastic cover is depicted. The design of the system is ready to use on-site. The electronic shield is optimized for proper changes to the measuring aperture from a cuvette to a 2 mL test tube. Thus, future developments on integration with flow methods or bioreactors are possible. The operational time of OMD was 4 h of consecutive streaming with 1 line of data per second. This acquisition rate could be raised if OMD is connected to a computer and, e.g., Data Streamer is employed to gather raw data.

### 3.2. Characterization of the Light Source

**Figure 6:**
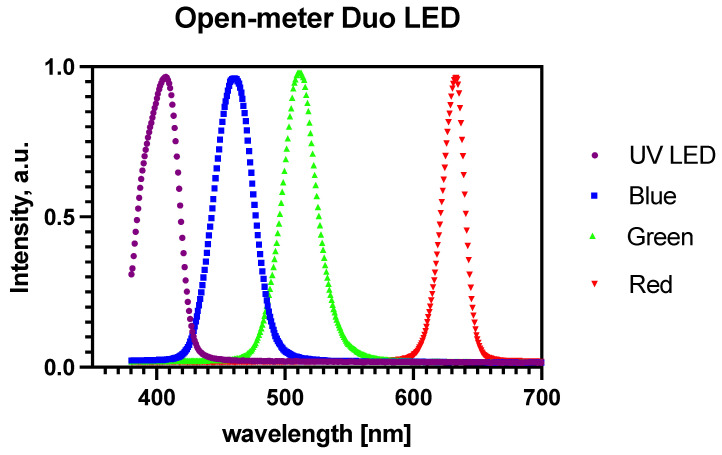
Emission spectra of OMD light source.

The characterization of the light source and response test of the sensor was conducted. Figure 6 shows the emission maxims of the light sources used. The maximas (with the full width at half maximum) were 405 nm for the UV LED (384–419 nm); 630 nm for the red LED (620–641 nm); 510 nm for the green LED (496–527 nm); and 460 nm for the blue LED (441–478 nm).

### 3.3. Response of Sensor

**Figure 7:**
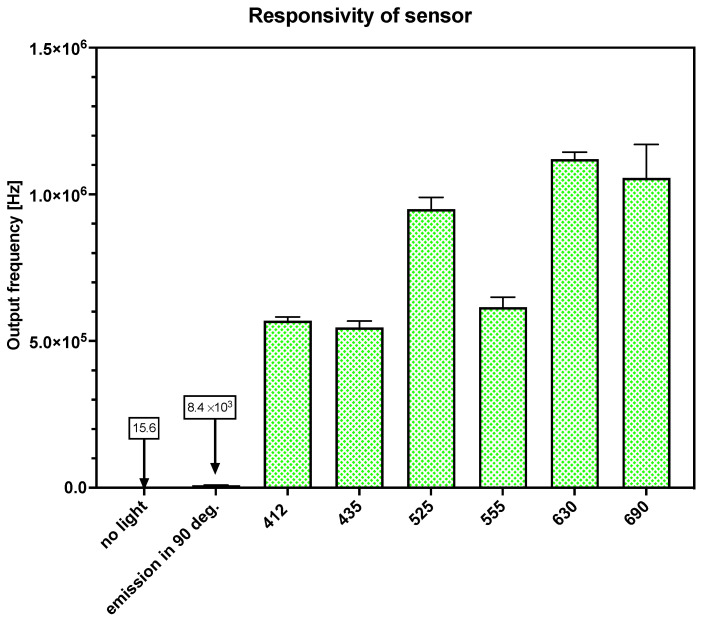
Comparison of TSL230R sensor’s response in different light conditions. Values on Y-axes indicates the response of the sensor when RGB LED imitating color of the chosen wavelength, the excitation light source was turned ON, or both light sources were turned OFF.

The response of the light-frequency sensor, TSL230R, was also evaluated with different imitated colors. The highest response to the activated light source was at least 0.5 MHz (light of different colors was simulated with distilled water in a cuvette; see Figure 7). The highest response was acquired with an imitated color of 690 nm. The lowest response was obtained with an imitated color of 435 nm. According to the datasheet of TSL230R, the sensor response should be highest at the edge of the visible and infrared part of the spectrum [31].

Thus, as the primary reason for developing the system presented here is to create the possibility of the field analysis of cholinesterase in the environment, we measured the response of the sensor in dark conditions. The resulting darkness in the box was sufficient, due to the mean sensor response being 15.5 Hz in the condition without light. Additionally, the intensity of the parasitic light from the excitation source was determined. The output frequency was 8.3 × 10^3^ Hz, while the sample value equaled 200. The response rate was very fast (approx. 0.5 ms) with CV 10.1%. Although this error is relatively high, it does not comprise the planned use of this low-cost instrument.

### 3.4. Remote Control Interface for Open Meter Duo

**Figure 8:**
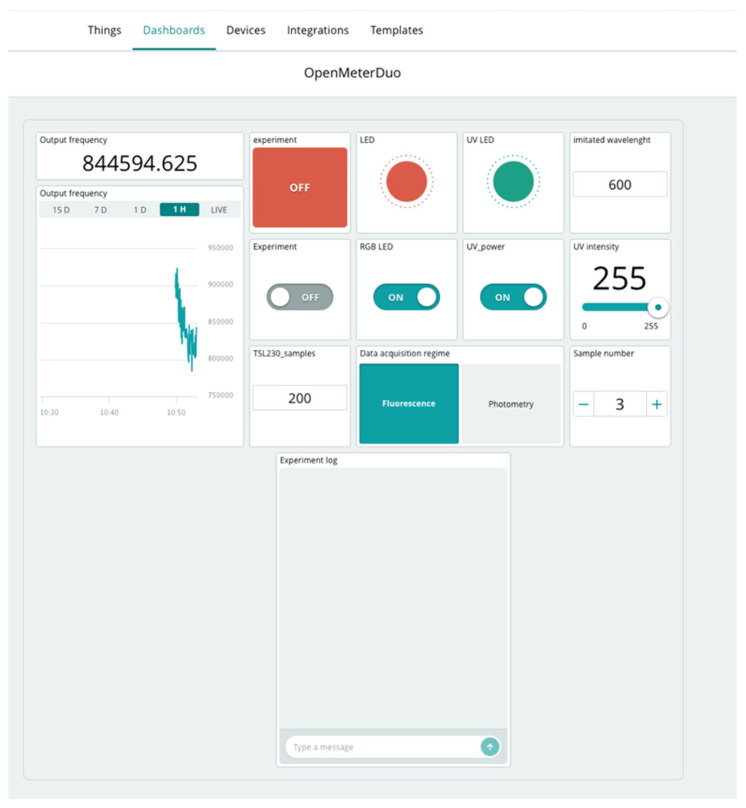
Overview of the example dashboard made for remote version of Open Meter Duo.

The Arduino cloud interface was used to was used to design the dashboard for remote data acquisition with OMD (see Figure 8).

The control commands programmed on the Arduino cloud interface were as follows:

- Experiment: ON/OFF.
- Messenger—experiment marking—time stamp to distinguish experiments.
- NumberOfSamples—counter of various experimental samples based on user demand.
- Switch between fluorimetry/photometry (with automatic change of sample value). It was necessary to manually switch the light source on for fluorimetry or photometry using the button.

At the end of measurements, it is possible to export the values of given variables in the last investigated period (up to 15 days). The values are exported to separated files. Users can choose the variable that is needed. In addition, exports can be conducted with the web app alone. The response rate for periodical acquisition on Arduino cloud is 1 value per second.

### 3.5. Indoxyl Acetate

#### 3.5.1. Spectral Characteristics of Indoxyl Acetate and Its Hydrolysis

**Figure 9:**
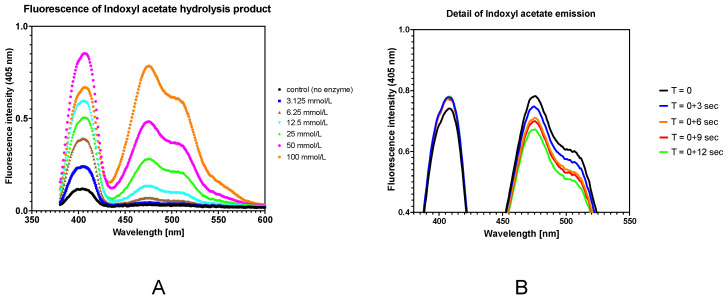
(**A**) Emission spectra of various concentrations of indoxyl acetate. (**B**) Comparison of fluorescence stability during real-time measurement.

In this work, we evaluated the wavelength where the fluorescence of the hydrolyzed indoxyl acetate occurred. As can be seen in Figure 9A, it was 404 and 475 nm, which corresponded with already-published works [4,41]. However, we found that the signals in the area of 480 nm did not have optimal behavior for kinetic measurements, because they were not stable. As can be seen in Figure 9B, the signal was slightly elevated in the first maximum after 3 s and was therefore still stable. The behavior of the second maxima was the opposite. The signal decreased after 12 s of testing.

#### 3.5.2. Simple Fluorimetric Approach

**Figure 10:**
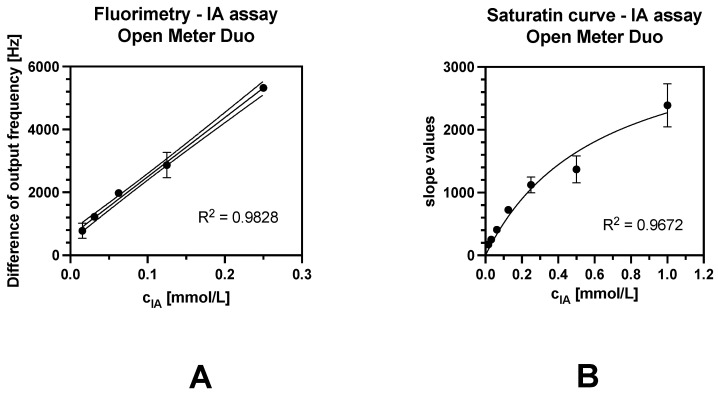
Calibration curve of indoxyl acetate. (**A**) Detail of end-point analysis; (**B**) evaluation of slope from isolated linear trends. The error bars indicate the standard error for n = 3.

A saturation curve for fluorimetric indoxyl acetate was constructed (see Figure 10). The kinetic parameters of the enzyme reaction were calculated based on the Michaelis–Menten model. Km of indoxyl acetate was 0.544 mmol/L in the evaluation of the kinetic assay. The limit of detection of indoxyl acetate was 11.8 μmol/L, which was determined using the difference in the output frequency approach (Figure 9A), and 65.4 μmol/L was determined using the slope evaluation approach (Figure 9B). This indicates that using slope values will not be very accurate when measuring samples with low cholinesterase activity. The results of the end-point fluorimetry are comparable to those of the stand-alone fluorimetry conducted by Navas Díaz et al., who determined the limit of detection for indoxyl acetate to be 13.8 μmol/L [41]. The measuring conditions were very similar in the first 100 s of the reaction to the first 120 s in our work.

In their work, Válek et al. tested indoxyl acetate for the photometric determination of lipase activity with the obtained detection limit of 92 μmol/L [42]. The detection limit was obtained by the evaluation of slope values obtained using this proposed system. However, the limits acquired by Válek et al. were determined from the 30 min reaction, which was the longest assay time.

#### 3.5.3. Study of Fluorescence Stability

**Figure 11:**
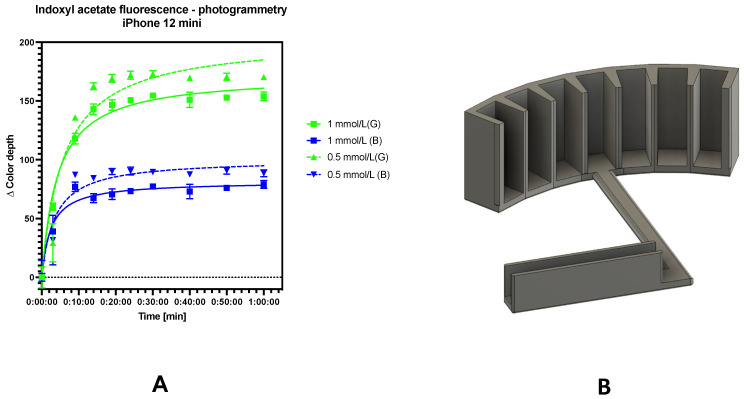
Stability test of the “white” fluorescent by-product of hydrolysis. Two concentrations of indoxyl acetate were used. Color intensity in the green and blue RGB channel was evaluated by photogrammetry (**A**) using a 3D-printed cuvette holder (**B**) placed in a dark chamber with a UV lamp. The error bars indicate the standard error for n = 3.

We found that the evolved fluorogenic product was stable at least after 1 h, since the fluorescence intensity was not decreased (based on photogrammetry under the UV lamp; see Figure 11A), which corresponds with previous research that indicates that indoxyl should be a stable fluorescent agent in pH < 7, without the oxidation of indoxyl to indigo [16]. Thus, fluorometric assays could be a promising tool for determining lipase activity, since the fluorescence signal is stable for at least 1 h, as this study on AChE was carried out using a lab-made cuvette stand for photogrammetry (see Figure 11B). The green channel is suitable for photogrammetry under UV conditions.

#### 3.5.4. Determination of Model Cholinesterase Inhibitor

Donepezil represents a drug used for the treatment of Alzheimer’s disease, a neurodegenerative disorder [5]. Analytical methods to study cholinesterase activity are usable in the development of novel molecules.

Table 2 shows IC_50_ values, which indicate the concentration of an inhibitor that lowers the activity of AChE to 50%, and also shows limits of detection in comparison with other works.

**Figure 12:**
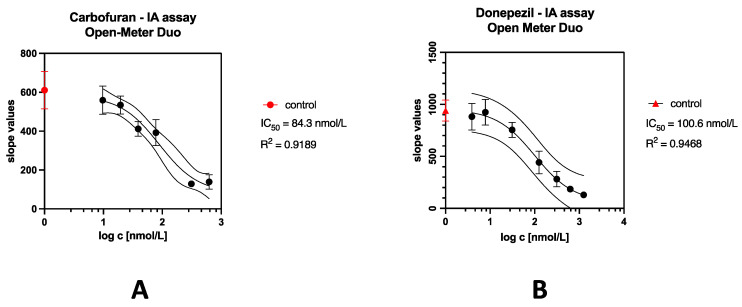
Calibration curves of cholinesterase inhibitors. (**A**) Carbofuran. (**B**) Donepezil. The error bars indicate the standard error for n = 3.

From the results shown in Figure 12, a visible trend in the dose–response curve could be seen. Thus, the assay is suitable for screening novel molecules with similar kinetics, or even monitoring the similarity of medicine quantity in herbal material.

The analysis of model compounds such as carbofuran and donepezil was conducted as alternative approach to other techniques. More optimization and tests of other pollutants inhibiting cholinesterase (such as organophosphate inhibitors and other Alzheimer’s disease medicines) are planned. It is possible that the future optimization of data analysis, for example, by employing artificial intelligence to properly find optimal reaction times, may lower the detection limit.

Although the obtained detection limits were bigger than in other works (see Table 2), this system could be still used in the field evaluation of herbal extracts’ effects on cholinesterase to make decisions based on inhibitory potential [9].

While sensors based on different principles are available for substances to be determined which achieve good detection limits, their principle will determine the final application. In the fluorimetric determination of cholinesterase, most determinations are based on flat platforms. In photographic determinations, we distinguish the color depth of the signal in the range of 0–255. However, as can be seen from Figure 11A and other photogrammetric works, the usual dynamic range of color depth in the analyzed color channel is between 50 and 150 [14,15,17].

#### 3.5.5. Dual-Signal Approach for Indoxyl Acetate Assay

**Figure 13:**
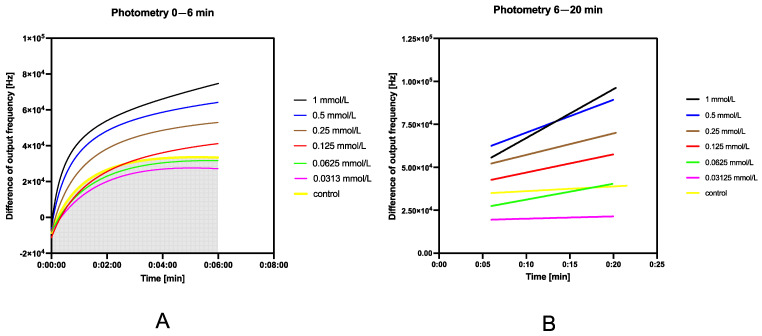
Dual-signal overview of indoxyl acetate assay. (**A**) Photometry—6 min assay after switching from fluorimetry. A non-linear change in the signal is evident. (**B**) Photometry—from 6 to 20 min of analysis, the signal has a linear trend of increase, which is the desired response of the experiment.

A dual-signal approach to cholinesterase determination was also demonstrated. A non-linear increase could be observed from 0 to 6 min during photometry; therefore, there was a linear trend in the interval between 6 and 20 min (see Figure 13A,B). When fluorimetry was coupled with photometry, an interesting dataset emerged. Noteworthily, the responses in this comparison were subtracted and inverted for better evaluation. The evaluation should employ artificial intelligence to process the data and to control the measurements during the evaluation.

Due to the large signal change within the first 6 min of photometric determination immediately after the fluorimetric measurement was conducted, it is evident that the air-oxidized conversion of indoxyl to indigo was put on hold by the UV light emitted during fluorimetric monitoring.

The 5 min observations seemed sufficient to determine the changing reaction trend if the fluorimetric regime was used solely. The evolution of the blue pigment indigo depended on the air-oxidation of the high-fluorescence product of indoxyl acetate hydrolysis—indoxyl [41,44].

Unless the supposed wavelengths for indoxyl acetate assay were 670 [8], 605 [37], or 650 nm [38], 630 nm was suggested also [12]. Due to long time it took the colored pigment to evolve, the photometric approach was used only for the visualization of the dual-signal use of this photometer. Based on the principle of transforming the wavelength to RGB format, it did not matter what wavelength was chosen as much as in spectrophotometry. The red channel would be at full intensity at all considerable wavelengths for traditional photometry. This is a considerable limit of OMD; thus, it is a significant issue if other absorbing compounds are present in the assay. However, when this occurs, Ellman’s assay could be conducted as well [33].

Photometric determination by the indoxyl acetate method is considered rather slow [13]. On the other hand, a significant benefit of the indoxyl acetate assay is that the substrate is either fluorogenic/chromogenic itself and it is more stable than Ellman’s reagent [8,16]. Future coupling of the tested procedures should be carried out with artificial intelligence cooperation in the evaluation of kinetics parameters in real time and should enable automatic decision making while performing ongoing analyses.

It may be interesting to use a color sensor instead of a light intensity sensor [45,46], either using a microcontroller or a single-board computer [30]. The use of color sensors may be advantageous, especially since fluorescence can be captured by tracking changes in the green color channel, as shown in our stability study. The colored product of indoxyl acetate hydrolysis can therefore be determined by the difference in red channel intensity, which has already been verified [11]. Admirable research has been carried out regarding the testing of color sensors and implementing these in low-cost analytical systems [45,46]. The resolution of the system based on color sensors is 16-bit, and the intensity of color depth is between 0 and 65,535 for each channel in RGB. In spite of fact that photogrammetry only has 8-bit resolution, and it measures color depth values of 0–255 for each color channel, photogrammetric assays have a place in end-point field assays rather than in kinetic assays. The reason for this is simple: data acquisition and evaluation. Additionally, the lower the resolution of the raw signal, the worse the chances are for further statistical evaluation without unreasonable errors in the first place.

Using a low-cost instrument based on photometry or colorimetry could create advantages for the future implementation of these concepts in agricultural or point-of-care testing technologies [47].

Working with datasets as large as the one shown in the figure above is quite complicated and requires the involvement of artificial intelligence, at least to speed up the evaluation and pre-evaluation. Although OMD can produce an interesting dataset in a coupled environment, it is not technically well suited for artificial intelligence involvement.

For example, RaspberryPi computers or Micropython-compatible microcontrollers with a parallel computation core would be ideal for this application.

## 4. Conclusions

This work proposes a novel, low-cost photo/fluorimeter, Open Meter Duo. It consists of Arduino UNO R4 and a novel prepared shield. This PCB shield employs a light-frequency sensor, TSL230R, a color-programmable RGB LED, and a UV LED. The fluorometric indoxyl acetate assay was tested by this system. The results showed that the OMD system can measure cholinesterase remotely with indoxyl acetate fluorimetrically and photometrically, which is not a widely used approach. We found the edge of the capabilities of using a microcontroller for the determination of cholinesterase with a low-cost sensor of light. Further work should concentrate on either color sensor-based photometry or small spectrometer-based systems. However, we expect that this method will have practical applicability in measuring cholinesterase in field conditions for the detection of neurotoxic pesticide presence. At this time, the system could be further used for drug control, point-of-care testing, and similar purposes where the portability and very low price of the device are substantial factors in choosing a proper method.

## Figures and Tables

**Table 1:** Bill for the materials used for the construction of Open Meter Duo. The currency is noted in USD.

Component	Number	Cost per Unit	Total Cost	Source of Materials	Material Type
One-sided positive pre-sensitized PCB card	1	USD 2	USD 2	https://www.amazon.com/Aoje-Link-Single-Copper-Laminate-Circuit/dp/B091T93HKS/ (accessed on 20 January 2024)	Composite
Arduino UNO R3/R4 Wi-Fi	1	USD 24 (offline) / USD 25 (remote)	USD 24 (offline) / USD 25 (remote)	https://store.arduino.cc/products/arduino-uno-rev3 /https://store.arduino.cc/products/uno-r4-wifi (accessed on 20 January 2024)	Semi-conductor
Header 4 × 1, 2.54 mm, female, straight	1	USD 1	USD 1	Local hardware store	Metal
Header 7 × 1, 2.54 mm, female, straight	1	USD 1	USD 1	Local hardware store	Metal
Male header 20 × 1, 2.54 mm, angle	1	USD 1	USD 1	Local hardware store	Metal
Male header, 20 × 1, 2.54 mm, straight	1	USD 1	USD 1	Local hardware store	Metal
TSL230R light-frequency sensor	1	USD 28 (2022) USD 51 (2024)	USD 28 (2022) USD 51 (2024)	https://www.aliexpress.com/item/1005006332337743.html (accessed on 20 January 2024)	Semi-conductor
RGB LED common cathode, diffuse	1	USD 1	USD 1	Local hardware store	Semi-conductor
Jumper wires	5	USD 0.2	USD 1	Local hardware store	Metal
Printing material (ASA)	100 g		USD 4	https://www.prusa3d.com/product/prusament-asa-signal-white-850g/ (accessed on 20 January 2024)	Polymer
Capacitor 0.1 μF	1	USD 1	USD 1	Local hardware store	Composite
Socket 4 × 2, DIP	1	USD 1	USD 1	Local hardware store	Other
UV LED from Centropen secure kit	1	USD 1	USD 1	https://www.amazon.de/dp/B01AUI4VTM (similar emission, accessed on 20 January 2024))	Semi-conductor
Battery holder for 4 × AAA	1	USD 2	USD 2	https://www.amazon.com/Pack-AA-AAA-Battery-Holders/dp/B08Q3DLY39 (accessed on 20 January 2024)	Other
Screw terminal 3-pin	1	USD 1	USD 1	Local hardware store	Other
Cable 2-wire (black, red; 50 cm/approx. 2 ft)	1	USD 1	USD 1	Local hardware store	metal

**Table 2:** Comparison of inhibition parameters of cholinesterase inhibitors. Donepezil hydrochloride and carbofuran. IC_50_ and LOD were evaluated. IA - Indoxyl acetate.

Analyte	Principle	Method	IC_50_ [nmol/L]	LOD [nmol/L]	Ref.
Donepezil	Spectrofluorimetry	IA assay	80.59	33.7	This work
	Fluorimetry (OMD)		100.6	54.45	
	Photogrammetry	Dipstick pH-metry	---	22.3	[43]
Carbofuran	Spectrofluorimetry	Ellman assay	102.9	17.15	[33]
	Fluorimetry (OMD)	IA assay	84.30	94.42	This work
	Photogrammetry		---	22.55	[14]

## Data Availability

The raw data supporting the conclusions of this article will be made available by the authors on request.
